# 

**Published:** 1903-09

**Authors:** 


### BOOKS RECEIVED.

The Latin Grammar of Pharmacy and Medicine. By D. H. Robinson, Ph. D., late Dean of School of Arts, and Professor of Latin Language and Literature, University of Kansas. With an introduction by L. E. Sayre, Ph. M., Professor of Pharmacy in, and Dean of, Department of Pharmacy, University of Kansas. Fourth edition. Thoroughly revised by Hannah Oliver, A. M. Philadelphia: P. Blakiston's Son & Company. 1903. (Price, $1.50.)

The Principles of Obstetrics. A Practical Manual for the Student and General Practitioner. By Stanley Perkins Warren, M. D., Portland, Me., Obstetric Surgeon to the Maine General Hospital. Octavo; 385 pages. Illustrated by 165 line and half-tone engravings. William Wood & Company, New York. 1903. (Cloth, $3.00; leather, $3.75 net.)

A Manual of Obstetrics. By A. F. A. King, M. D., Professor of Obstetrics and Diseases of Women, in the Medical Department of the Columbian University, Washington, D. C., and in the Medical Department of the University of Vermont. Ninth edition, revised and enlarged. In one 12mo volume of 628 pages, with 275 illustrations. Lea Brothers & Co., Philadelphia and New York. 1903. (Cloth, $2.50 net.)

Plain Hints for Busy Mothers. By Marianna Wheeler, Superintendent of the Babies' Hospital, New York, and author of The Baby. E. B. Treat & Co., New York. 1903. (Price, 35 cents.)

Radium and other Radioactive Substances. With a Consideration of the Treatment of Disease by the Ultraviolet Light. By William J. Hammer, Consulting Electrical Engineer. 12mo., pp. 72. Illustrated. New York: D. Van Nostrand Co. 1903. (Price, $1.00.)

The Practical Medicine Series of Year Books. Comprising ten volumes on the year's progress in Medicine and Surgery. Issued monthly under the general editorial charge of Gustavus P. Head, M. D., Professor of

Laryngology and Rhinology in the Chicago Post-Graduate Medical School. Volume VII. Pediatrics. Edited by Isaac A. Abt, M. D., Assistant Professor of Medicine, Rush Medical College. Orthopedic Surgery. Edited by John Ridlon, M. D., Professor of Orthopedic Surgery, Northwestern University Medical School. Chicago: The Year Book Publishers. 1903. (Price, $1.25; entire series, $7.50.)

